# Genomic characterization and virulence of *Streptococcus suis* serotype 7 sequence type 373 of clonal complex 94

**DOI:** 10.1186/s13567-025-01562-4

**Published:** 2025-06-23

**Authors:** Rujirat Hatrongjit, Parichart Boueroy, Peechanika Chopjitt, Thidathip Wongsurawat, Piroon Jenjaroenpun, Natnicha Wankaew, Zeren Peng, Han Zheng, Marcelo Gottschalk, Zongfu Wu, Anusak Kerdsin

**Affiliations:** 1 Department of General Science, Faculty of Science and Engineering, Kasetsart University, Chalermphrakiat Sakon Nakhon Province Campus, Sakon Nakhon, 47000 Thailand; 2 Faculty of Public Health, Kasetsart University, Chalermphrakiat Sakon Nakhon Province Campus, Sakon Nakhon, 47000 Thailand; 3 Siriraj Long-Read Laboratory, Division of Medical Bioinformatics, Department of Research and Development, Faculty of Medicine Siriraj Hospital, Mahidol University, Bangkok, Thailand; 4 WOAH Reference Lab for Swine Streptococcosis, MOE Joint International Research Laboratory of Animal Health and Food Safety, College of Veterinary Medicine, Nanjing Agricultural University, Nanjing, China; 5 State Key Laboratory of Infectious Disease Prevention and Control, National Institute for Communicable Disease Control and Prevention, Chinese Center for Disease Control and Prevention, Beijing, China; 6 Research Group on Infectious Diseases in Production Animals (GREMIP), Faculty of Veterinary Medicine, University of Montreal, Quebec, Canada; 7 Affiliated Researcher at the Faculty of Veterinary Science, Chulalongkorn University, Bangkok, 10330 Thailand; 8 Center of Excellence for Food and Water Risk Analysis (FAWRA), Department of Veterinary Public Health, Faculty of Veterinary Science, Chulalongkorn University, Bangkok, 10330 Thailand

**Keywords:** *Streptococcus suis*, ST373, serotype 7, antimicrobial resistance gene, genomic characteristic, virulence

## Abstract

*Streptococcus suis* is a swine pathogen that also causes invasive infections in humans, leading to significant economic losses in pig production worldwide. Serotype 2 is the most pathogenic *S. suis* strain associated with human infections. However, non-serotype 2 strains isolated from humans have been reported globally. Here, we conducted a comparative genomic analysis of *S. suis* serotype 7-ST373 strains belonging to clonal complex 94, isolated from both humans and pigs, and assessed their virulence through mouse infection experiments. Genomic analysis revealed that *S. suis* serotype 7-ST373 strains harbor genomic islands 1–3 of the pathogenic *S. suis* clade. They also possess a high number of virulence-associated genes, similar to those of virulent serotype 2 strains, suggesting a high virulence potential. The antimicrobial resistance gene *tet(O)*, which confers tetracycline resistance, was found in all ST373 strains, while *erm(B)*, which confers macrolide resistance, was detected in most ST373 strains. However, the macrolide and lincosamide resistance genes *lnu(B)* and *lsa(E)* were found exclusively in a Thai human strain. Comparative genomics of ST373 strains with the virulent serotype 2 strain P1/7 identified 76 unique genes in ST373 strains, including 30 genes exclusively present in human ST373 strains. Mouse virulence experiments with two human ST373 strains (GX69 and STC2826) and one strain from a healthy pig (WUSS318) resulted in 100% mortality, classifying them as highly virulent. These findings indicate that serotype 7-ST373 strains demonstrate pathogenic potential and should be closely monitored.

**Supplementary Information:**

The online version contains supplementary material available at 10.1186/s13567-025-01562-4.

## Introduction

*Streptococcus suis* is a zoonotic pathogen that causes invasive infections in both humans and pigs [1]. Among the 29 known serotypes, serotypes 2 and 14 are the most frequently isolated from human *S. suis* infections. However, other serotypes, including serotypes 1, 4, 5, 7, 9, 16, 21, 24, and 31, have occasionally been recovered from human cases [1–3]. Serotype 7 is commonly reported in diseased pigs from Europe, North America, and Thailand [1, 3–5]. This serotype has been associated with severe diseases, such as meningitis and septic arthritis, particularly in nursery and grower pigs within affected herds [4, 5].

The first human case of *S. suis* serotype 7 was reported in China in 2021, following its isolation in 2016 [3]. This strain was identified as sequence type (ST) 373 and was shown to be virulent in a mouse infection model [3]. ST373 belongs to clonal complex (CC) 94 [6]. However, previous studies did not analyse the genomic characteristics of CC94, particularly the ST373 population among human and pig isolates [3].

Here, we report the second known human infection caused by serotype 7-ST373. We describe a comparative genomic analysis of *S. suis* ST373 strains isolated from humans and pigs, providing insights into their genomic characteristics, putative virulence genes, and genetic relationships.

## Materials and methods

### Bacterial strains, identification, and antimicrobial susceptibility

The *S. suis* serotype 7 strain STC2826 used in this study was isolated from a human blood sample in Lampang Province, Thailand, in 2023. The strain was cultured on sheep blood agar, and DNA was extracted using the ZymoBIOMICS DNA Kit (Zymo Research, CA, USA) following the manufacturer’s instructions. Species confirmation and serotyping of all *S. suis* strains were performed using previously described PCR assays [7].

The minimum inhibitory concentration (MIC) for penicillin, ampicillin, ceftriaxone, meropenem, daptomycin, azithromycin, erythromycin, tetracycline, and levofloxacin was determined using the Liofilchem^®^ MIC Test Strip (Liofilchem, Italy) according to the manufacturer’s instructions. Since no *S. suis*-specific breakpoints are available, MIC results were interpreted using the Clinical and Laboratory Standards Institute (CLSI) guidelines M100 (33rd edition) for viridans group streptococci [8]. *Streptococcus pneumoniae* strain ATCC 49619 was used as a quality control strain.

### Whole-genome sequencing

The genome of strain STC2826 was sequenced using the Oxford Nanopore Technologies (ONT) platform. Library preparation followed the rapid barcoding DNA sequencing protocol (SQK-RBK004 kit) without DNA size selection to retain plasmid DNA. Sequencing was performed using a MinION™/GridION™ flow cell (R10.4.1) on a GridION sequencer. Base calling and demultiplexing of raw data were conducted using Guppy v3.4.5 (ONT), and ONT adapters were trimmed using Porechop v0.2.4 [9]. Quality control of ONT reads was performed using Nanoplot v1.28.1 [10]. Genome assemblies were generated using Unicycler v0.4.8 [11], and genome quality was assessed using QUAST v5.0.2 [12]. Genome annotation was performed using the NCBI Prokaryotic Genome Annotation Pipeline (PGAP v4.12). Unless otherwise specified, default parameters were used for all software. The genome sequence of *S. suis* serotype 7 strain STC2826 was deposited in NCBI GenBank under BioProject accession number PRJNA691075.

### Bioinformatics analysis

ST classification was confirmed using the PubMLST database [13]. Antimicrobial resistance genes and plasmid replicons were identified using ResFinder 4.1 [14] and PlasmidFinder 2.1 [15]. Mobile genetic elements were detected using ICEfinder [16]. The presence of 106 virulence-associated genes (VAGs; Additional file 1) and genomic islands 1–3, previously associated with *S. suis* virulence or the pathogenic clade, was screened using MyDbFinder 2.0 [6, 17, 18]. Genes with > 90% coverage matches and > 85% identity matches were classified as present.

A dataset of 96 curated *S. suis* CC94 genomes [19] was analysed alongside the serotype 7-ST373 genome generated in this study to construct a phylogenetic tree of the CC94 population (Additional file 2). Phylogenetic analysis was performed using a reference genome-based single-nucleotide polymorphism (SNP) approach with REALPHY [20], and the resulting tree was visualized using iTOL v4 [21]. The *S. suis* serotype 2 reference strain S735 (accession no. CP003736) was used as the reference genome for SNP analysis.

Pangenome analysis was conducted using the anvi’o v7 workflow [22]. This workflow identified gene clusters and single-copy genes across the studied genomes, including all ST373 strains and two serotype 2 genomes: the epidemic strain SC84 (Accession no. FM252031) and the highly virulent strain P1/7 (Accession no. AM946016). The pangenome was divided into core and accessory bins based on gene cluster frequency. Gene overlap among bacterial strains was visualized using UpSetR, implemented in R [23].

### Mouse infection experiments

Animal infection experiments were conducted at the Laboratory Animal Center of Nanjing Agricultural University under Permit Number SYXK (Su) 2021–0086. The study, including animal experiments, was reviewed and approved by the Experimental Animal Welfare and Ethics Committee of Nanjing Agricultural University. Five-week-old BALB/c mice were purchased from Shanghai SLAC Laboratory Animal Co., Ltd. (Shanghai, China). The selected *S. suis* ST373 strains included STC2826 (a human isolate from Thailand), GX69 (a human isolate from China), and WUSS318 (an isolate from a healthy pig in China). Control groups included the highly virulent serotype 2 strain SC070731 [24], the non-virulent serotype 9 strain SH040917 [25], and a phosphate-buffered saline (PBS) control. Each experimental group consisted of 10 mice, with each mouse receiving an intraperitoneal injection of 3 × 10^8^ CFU. Survival was monitored for seven days post-infection. Based on prior studies [3, 26, 27], a strain was classified as highly virulent if it resulted in a mortality rate of ≥ 80% in infected mice. Statistical analysis was performed using the Log-Rank (Mantel-Cox) test.

## Results

### *Streptococcus suis* ST373 population

**Figure 1:**
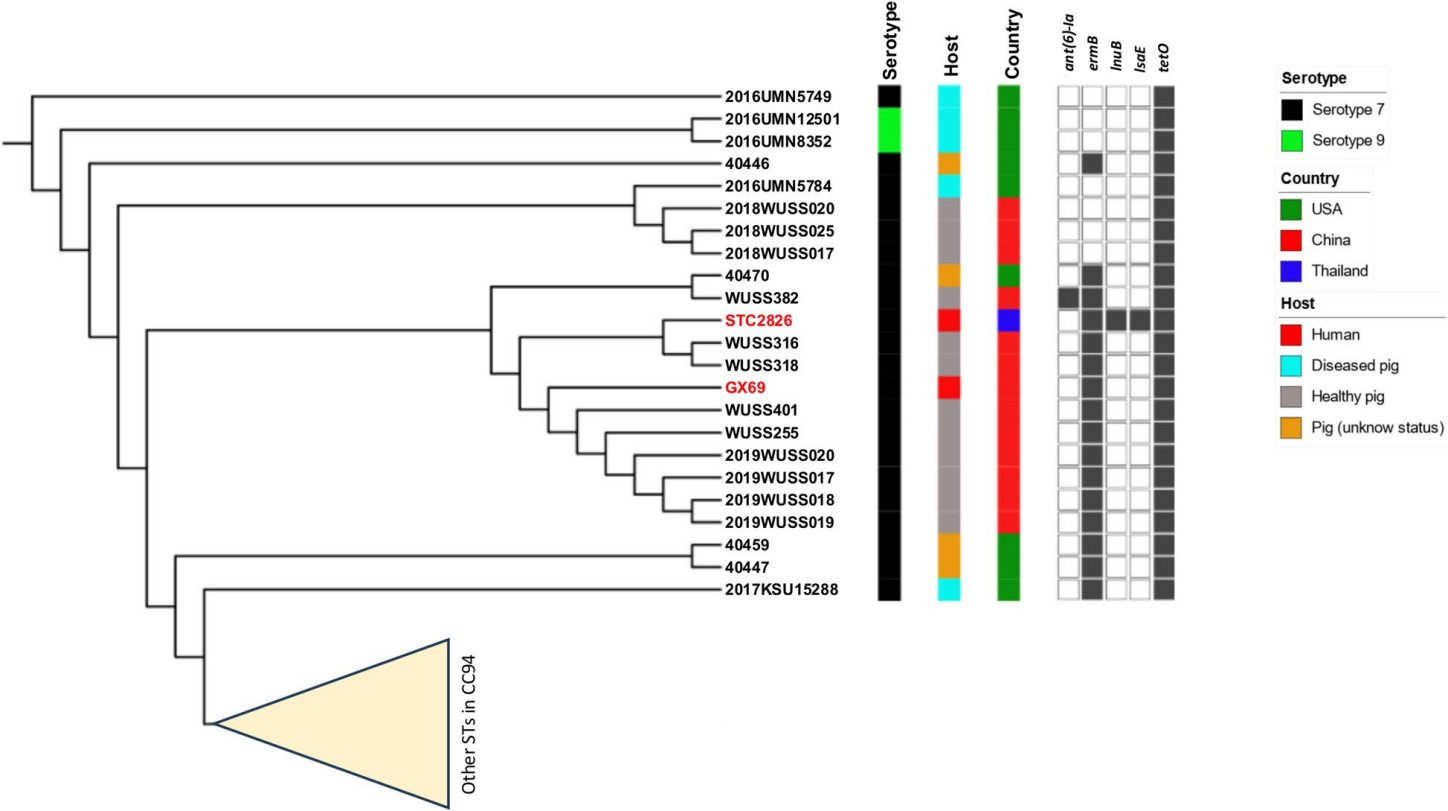
**Whole-genome SNP reference mapping-based phylogeny of *Streptococcus suis* ST373, generated using REALPHY and visualized with the Interactive Tree of Life (iTOL) tool.** The clade of *S. suis* serotype 7-ST373 strains from this study and their closely related strains are shown along with their serotypes, sources of isolation, and countries of origin. Filled squares indicate the presence of antimicrobial resistance genes.

A Nanopore assembly of the *S. suis* serotype 7-ST373 strain STC2826 revealed a genome size of 2 403 924 bp, with an N50 value of 799 119 bp. This strain contained 2382 coding sequences, 58 tRNA genes, and 12 rRNA genes. The replicon type could not be identified using PlasmidFinder 2.1. Genome-based MLST analysis confirmed that STC2826 belonged to ST373 within CC94. The whole-genome SNP-based phylogeny of all ST373 genomes (*n* = 23) is shown in Figure 1. The STC2826 strain was closely related to healthy pig isolates from China (WUSS316 and WUSS318). Although the Chinese human strain GX69 was more distantly related to the Thai strain, STC2826 clustered with other Chinese strains (Figure 1), suggesting a shared ancestral origin.

### Antimicrobial resistance

The STC2826 strain was susceptible to penicillin (MIC = 0.023 µg/mL), ampicillin (MIC = 0.016 µg/mL), ceftriaxone (MIC = 0.064 µg/mL), meropenem (MIC = 0.006 µg/mL), daptomycin (MIC = 0.125 µg/mL), and levofloxacin (MIC = 0.25 µg/mL). However, resistance was observed against azithromycin (MIC > 256 µg/mL), erythromycin (MIC > 256 µg/mL), and tetracycline (MIC > 64 µg/mL).

ResFinder 4.1 identified the antimicrobial resistance genes *tet(O)* (tetracycline resistance), *lnu(B)*, *lsa(E)*, and *erm(B)*, which confer resistance to macrolide-lincosamide-streptogramin (MLS) antibiotics, in strain STC2826 (Figure 1). As shown in Figure 1, *tet(O)* was detected in all ST373 strains, while *erm(B)* was present in most ST373 strains. STC2826 carried additional resistance genes (*lnu(B)* and *lsa(E)*) not commonly found in other ST373 strains. Analysis of mobile genetic elements carrying antimicrobial resistance genes in STC2826 using ICEfinder revealed that *tet(O)* and *erm(B)* were located on a putative integrative and conjugative element (ICE) of 189 299 bp between positions 254 273 and 443 571 on the chromosome (Additional file 3).

**Figure 2:**
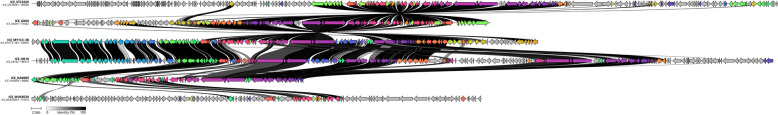
**Comparative analysis of integrative and conjugative elements (ICE) carrying** ***tet(O)*** **and** ***erm(B)*** **in** ***S. suis*** **strains STC2826, GX69, MY1C3-3B, HB18, HA0609, and WUSS030.**

Comparative analysis showed that this ICE was highly homologous to that of the Chinese human strain GX69 and shared similarity with ICEs from *S. suis* strains HB18 (pig, China), MY1C3-3B (pig, Canada), HA0609 (pig, China), and WUSS030 (pig, China). A comparison of ICE elements among STC2826, GX69, WUSS030, HB18, MY1C3-3B, and HA0609 is shown in Figure 2. Homologous regions were observed among STC2826, GX69, MY1C3-3B, HB18, and HA0609, whereas the ICE of WUSS030 differed. These findings suggest that the human ST373 strains STC2826 (Thailand) and GX69 (China) may have acquired ICE elements from a common origin. However, no mobile genetic elements carrying *lsa(E)* or *lnu(B)* were detected.

### Genomic comparison with serotype 2 virulent reference strain P1/7

**Figure 3:**
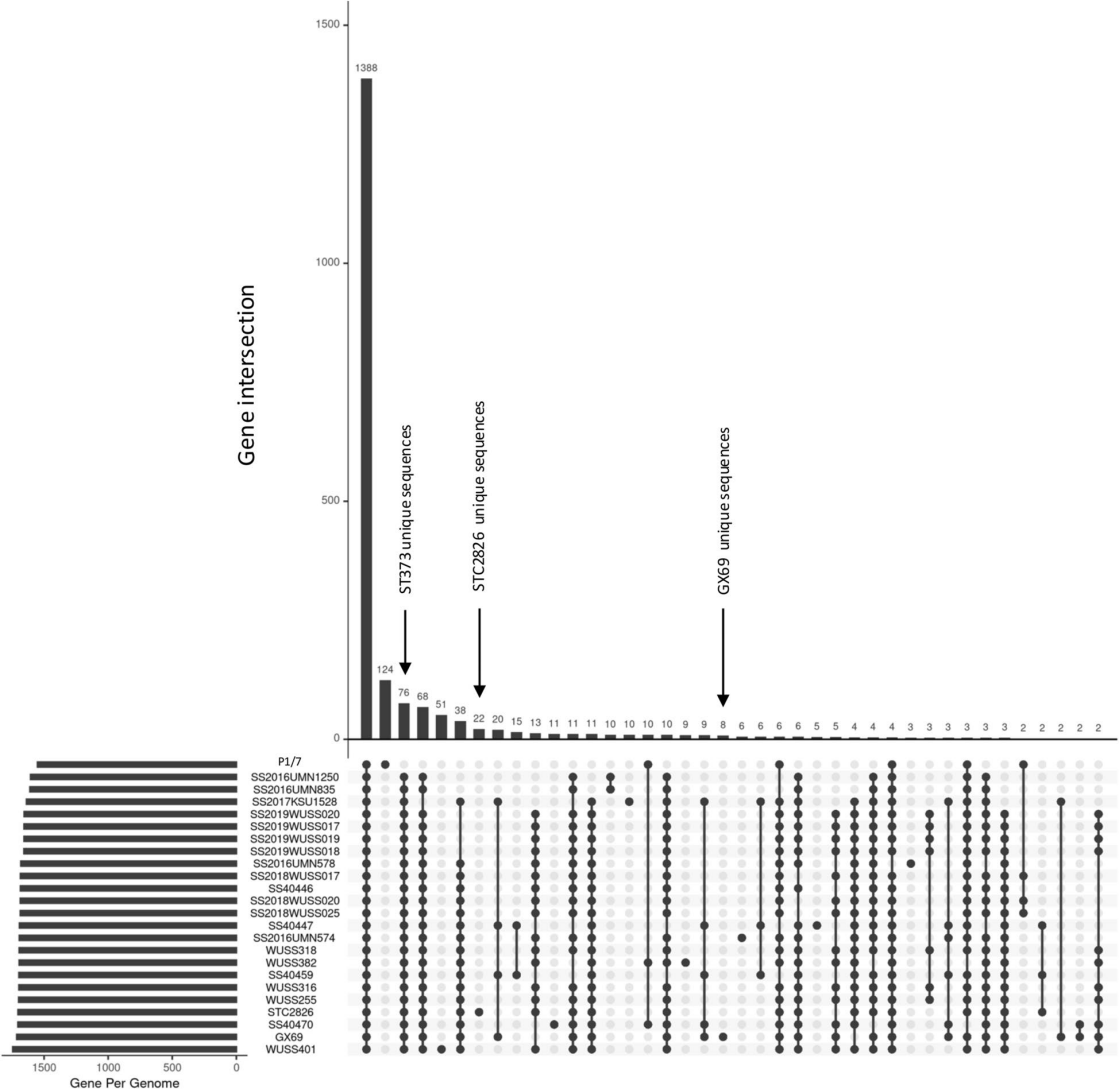
**Pangenome analysis of *S. suis* serotype 7-ST373 strains and serotype 2 strain P1/7, visualized using anvi’o.** The UpSetR plot represents the number of shared and unique genes among the analysed genomes.

A comparative genomic analysis was performed between all serotype 7-ST373 strains and the reference virulent serotype 2 strain P1/7 (ST1). A total of 1388 genes were shared between ST373 and P1/7 strains (Figure 3). The P1/7 genome contained 124 unique genes, whereas 76 genes were specific to ST373 strains. Additionally, 22 genes were found exclusively in the STC2826 strain, while 8 were unique to the GX69 strain (Additional file 4). Among the genes unique to serotype 7-ST373 strains, we identified those involved in carbohydrate, amino acid, lipid, nucleotide, coenzyme, and inorganic ion transport and metabolism, as well as genes related to energy production and conversion, secondary metabolite biosynthesis, cell wall/membrane/envelope biogenesis, signal transduction mechanisms, replication, recombination, and repair, transcription, the mobilome (prophages, transposons), defense mechanisms, and several genes encoded proteins of unknown function (hypothetical proteins) (Additional file 4).

Thai human serotype 7-ST373 strain STC2826 revealed 22 unique sequences which encoded site-specific DNA recombinases (SpoIVCA and XerD), DNA-binding transcriptional regulator (ArsR and MerR families), DNA repair and mobilization proteins (RecT, DnaD domain protein, and plasmid mobilization protein), proteins involved in metal resistance (CadD, CzcD) and antibiotic resistance (Lnu(B)), ATP-binding cassette (ABC) transporters, serine/threonine protein kinase, ubiquinone/menaquinone biosynthesis C-methylase UbiE/MenG, and hypothetical proteins. In contrast, the Chinese serotype 7-ST373 strain GX69 contained 8 unique sequences including transcriptional regulator contains XRE-family HTH domain, NAD-dependent DNA ligase, phage regulatory protein Rha, DUF1492 domain-containing protein, and hypothetical proteins. These 22 and 8 unique sequences of strains STC2826 and GX69 were summarized in Additional file 4.

### Pathogenic pathotype determinants

**Figure 4:**
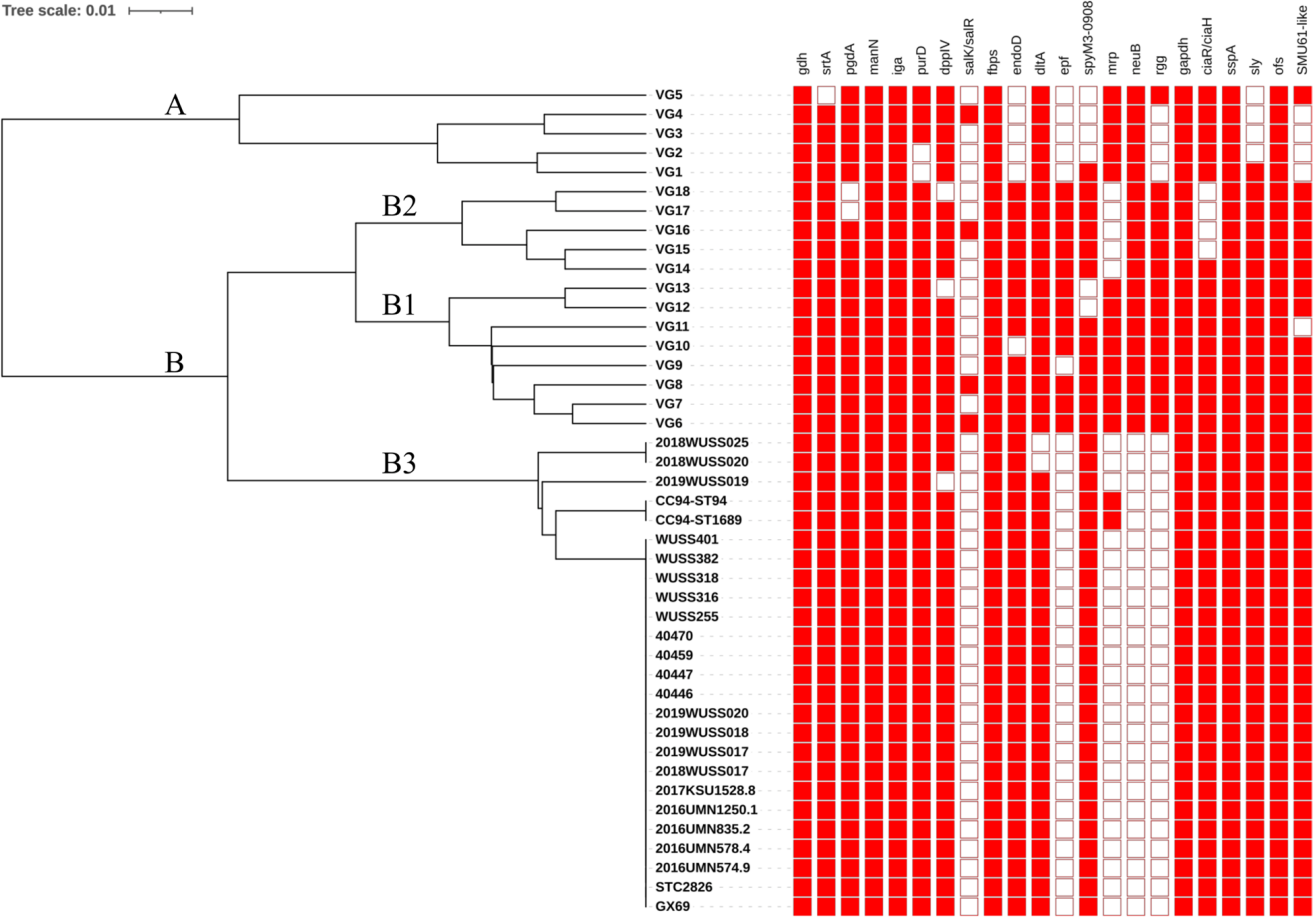
**Clustering of *S. suis* clonal complex 94 strains (subcluster B3) based on virulence-associated gene (VAG) profiles.** Filled squares indicate the presence of VAGs, while blank squares represent their absence. Clusters A and B (subclusters B1–B3) are labelled on the phylogenetic tree.

Analysis of 106 VAGs in all 23 ST373 strains showed that 26 VAGs including *cbp40omp40*, *epf*, *fhb-I*, *adhesin P*, *Hhly3*, *IgdE*, *mrp, nadR, neuB, NisK*, *NisR, pnuC, revS, rgg, salK, salR, sbp-1*, *sbp-2*, *srtB*, *srtC*, *srtD, srtG*, *SSU05-0473, virA*, *virB4*, and *virD4* were absent from all ST373 strains (Additional file 1)*.* CC94 encompasses diverse VAG profiles. The classical VAG profiles of all ST373 strains were *epf-/mrp-/sly* +. In addition, ST373 strains in this study carried six (30%) potential zoonotic virulence factors (PZVFs) in 21 PZVFs: *IdeS*, *rfeA*, *sly*, *SP1, tran,* and *zmpC* (Additional file 1). Additionally, 20 out of 23 strains contained *hylA*, which encodes hyaluronidase, whereas pig strains 2017KSU1528.8, 40447, and 40459 lacked this gene (Additional file 1). The ST373 isolates grouped in subcluster B3, along with ST94 and ST1689 of CC94 (Figure 4). Our analysis revealed that all ST373 isolates from both humans and pigs lacked four VAGs (*salK/salR*, *epf*, *neuB*, and *rgg*), similar to ST94 and ST1689 (Figure 4). Among the six VAGs commonly found in virulent serotype 2 cluster B strains, only *epf* and *rgg* were absent in ST373, ST94, and ST1689 within CC94 (Figure 4). Finally, most ST373 strains carried three genomic islands (GI-1–GI-3; similarity ≥ 85%) which is present in pathogenic *S. suis* clade, with the exception of strains 2017KSU1528.8, 40447, and 40459, which lacked GI-1.

### Mouse infections

To assess the virulence of ST373 strains, we selected three ST373 strains, one from a healthy pig (WUSS318) and two from human cases (GX69 and STC2826), for the mice infection experiments. All three ST373 strains exhibited a 100% mortality rate in mice, indicating that they are highly virulent (Table 1). The virulence of the human isolates GX69 and STC2826 did not significantly differ from that of the healthy pig-derived strain WUSS318.

**Table 1:** **Mortality rates of mice infected with** ***S. suis* ST373 strains**

Strains	Death in each period							Total death	Mortality rate (%)	*P*	Significance	Serotype
	1d	2d	3d	4d	5d	6d	7d					
GX69	7	3	0	0	0	0	0	10	100	/	/	7
STC2826	7	3	0	0	0	0	0	10	100	> 0.9999	ns	7
WUSS318	6	4	0	0	0	0	0	10	100	0.6477	ns	7
SC070731	10	0	0	0	0	0	0	10	100	0.0671	ns	2
SH040917	0	0	0	0	0	0	0	0	0	< 0.0001	****	9
PBS	0	0	0	0	0	0	0	0	0	< 0.0001	****	/

The virulence of strains was compared to strain GX69 using the Log-Rank (Mantel-Cox) test.

The symbols **** corresponds to *P* < 0.0001, while “ns” signifies no significance.

## Discussion

*S. suis* ST373 has been reported in swine from the United States, South Korea, China, and Thailand [3, 13]. However, human infections caused by ST373 were first reported in China [3], whereas the present study describes a case from Thailand. A previous study classified ST373 within lineage 3a [3]. Globally, *S. suis* strains from both human and pig sources exhibit high resistance to tetracyclines and macrolides (e.g., erythromycin and azithromycin) [28–31]. Our human strain STC2826 was also resistant to tetracyclines, erythromycin, and azithromycin due to *tet(O)*, *erm(B)*, *lsa(E)* and *lnu(B)* present in the chromosome. Several studies have reported that *tet(O)* and *erm(B)* are widespread among *S. suis* isolates from both pigs and humans across various serotypes [31, 32]. The *lsa(E)* and *lnu(B)* genes have been detected in *S. suis* serotypes 2 and 4 from human and pig isolates in Poland, Vietnam, China, Thailand, and the United Kingdom [6, 31, 33, 34].

The comparative genomic analysis among ST373 with a reference P1/7 strain revealed 22 and eight unique sequences in human strains STC2826 and GX69. These unique sequences involve in gene expression or regulation, DNA processing, transportation of metal ions or other substances, defense mechanism, signal transductions, and metaboic enzymes. Some of them are involved in bacterial virulence and stress response. In an example, *cadDX* operon contributes to oxidative stress resistance and virulence in both *S. suis* and *Streptococcus agalactiae* [35]. Serine/threonine protein kinase was also involved virulence, environment/host adaptation, and specific host defense processes [36]. ABC transporters play a vital role in the virulence and pathogenesis of several pathogenic bacteria through the import of essential molecules, such as metal ions, amino acids, peptides, vitamins and osmoprotectants, as well as, the export of virulent determinants [37]. We hypothesize that these unique sequences may be influence on these human strains to survive and adaptation in human body.

The classical VAG profiles of all ST373 strains were *epf-/mrp-/sly* +. A previous study showed that *mrp* and *sly* present in CC94 serotype 4 strains [6]. However, the ST373 strains analysed in this study, despite belonging to CC94, did not carry *mrp*, suggesting that CC94 encompasses diverse VAG profiles. Another study identified 21 PZVFs, including *cbp40omp40*, *fhb-I, adhesin P (fhb-II)*, *Hhly3*, *hyl*, *IdeS*, *IgdE, mrp*, *neuB*, *NisK, NisR*, *pnuC*, *rfeA*, *rgg*, *sbp-1, sbp-2, sly, sp1, tran, zmpC,* and *cps2BEFGJL*, which were predominantly found in CC1 strains [19]. In contrast, ST373 contained only six PZVFs (*IdeS, rfeA, sly, SP1, tran,* and *zmpC*). Previous studies suggested that PZVFs are more specific to CC1 than to other CCs [6, 19], implying that CC94, particularly ST373, may possess a distinct set of PZVFs.

A previous study analysing 22 VAGs (*gdh*, *srtA, pgdA, manN, iga, purD, dppIV, salK/R, fbps, endoD, dltA, epf, spyM3_0908, mrp, neuB, rgg, gapdh*, *ciaR/H, sspA, sly, ofs,* and *SMU_61-like*) in Chinese *S. suis* serotype 2 strains classified them into cluster A (low virulence) and cluster B (high virulence) [38]. Cluster B strains commonly harbored six VAGs (*epf, sly, endoD, rgg, SMU_61-like,* and *SpyM3_0908*), which are typically associated with virulent serotype 2 strains but are rarely found in cluster A strains [38]. All ST373 strains in this study were clustered in subcluster B3. This differential distribution of VAGs suggests that virulence-associated genes may vary among *S. suis* lineages.

A recent study identified three genomic islands (GI-1–GI-3) that are present in nearly all pathogenic *S. suis* clades [18]. Most ST373 strains carried GI-1–GI-3. This finding aligns with the VAG analysis, supporting the classification of ST373 as a virulent or pathogenic pathotype. Previous studies have reported that CC94 strains are associated with pathogenic or potentially virulent pathotypes [3, 6, 39]. Although CC94 predominantly comprises pathogenic isolates, some opportunistic and commensal strains have been identified [39]. In that study, serotype 7 was significantly associated with the pathogenic pathotype based on proportional analysis and odds ratio (OR) calculations [39]. Another study demonstrated that serotype 4-ST94 and ST1689 strains of CC94 exhibited potential virulence based on cell cytotoxicity assays [6]. Furthermore, previous research indicated that serotype 7-ST373 (lineage 3a) strains exhibited varying degrees of virulence in mouse models [3]. The detection of ST373 in both diseased and healthy pigs, as well as in humans, further supports its classification as a pathogenic pathotype [3].

We conducted mouse experiment with two human and one healthy pig strains of ST373 to prove their virulent. All mice were completely died in the experiment with these three strains. This indicated that ST373 are virulent according to their pathogenic determinants described above and suggesting that healthy pigs may serve as reservoirs for virulent strains capable of causing human infections. A previous study reported that mice infected with *S. suis* serotype 7 strains exhibited symptoms of sepsis, with survival rates varying among strains [3]. The survival rate of mice infected with strain GX69 declined sharply within 12 h post-infection, dropping to 10% by 24 h—closely resembling the virulence pattern of the highly virulent *S. suis* serotype 2 strain P1/7 [3]. In this study, the Thai human isolate STC2826 exhibited virulence comparable to that of the Chinese strain GX69. Moreover, prior research demonstrated that CC94 is highly pathogenic in both zebrafish and mice, with mortality rates exceeding 80% and is recognized as a pathogenic clone [39, 40]. Collectively, these finding highlighted that *S. suis* ST373 strains are highly pathogenic potential and capable of causing infection. The potential public health risk posed by *S. suis* serotype 7, particularly ST373 strains, warrants further surveillance and monitoring.

Comparative genomic analysis of *S. suis* serotype 7-ST373 strains isolated from human patients and pigs revealed the presence of numerous virulence-associated genes commonly found in the highly virulent serotype 2 strain P1/7. Additionally, ST373 strains carried genomic islands associated with the pathogenic *S. suis* clade, strongly suggesting a high pathogenic potential. Experimental infection in mice demonstrated 100% mortality, confirming their virulence. The identification of unique genes exclusively present in ST373 strains suggests a possible role in virulence or adaptation to specific environments within this lineage. Given their high virulence potential, *S. suis* serotype 7-ST373 strains should be closely monitored for their potential impact on public health and animal health.

## Supplementary Information

**Additional file 1**. **Virulence-associated genes present in *Streptococcus suis* ST373.**

**Additional file 2.** ***Streptococcus suis* clonal complex 94 genomes used to construct the phylogeny of the *S. suis* CC94 population.**

**Additional file 3.** **Predicted open reading frame of integrative and conjugative element detected in *Streptococcus suis* serotype 7 ST373 strain STC2826.**

**Additional file 4.** **Unique sequences of *Streptococcus suis* ST373 strains.**

## Data Availability

The datasets used and/or analysed during the current study are available from the corresponding author on reasonable request.
